# Untargeted metabolomics as a tool to assess the impact of dietary approaches on pig gut health: a review

**DOI:** 10.1186/s40104-025-01238-1

**Published:** 2025-07-22

**Authors:** Li-Hsuan Chen, Nuria Canibe, Mihai Victor Curtasu, Mette Skou Hedemann

**Affiliations:** Department of Animal and Veterinary Sciences, Aarhus University, 8830 Tjele, Denmark

**Keywords:** Dietary approach, Gut health, Piglets, Untargeted metabolomics

## Abstract

Metabolomics utilizes advanced analytical profiling techniques to comprehensively measure small molecules in cells, tissues, and biological fluids. Nutritional metabolomics studies in pigs have reported changes in hundreds of metabolites across various sample types, including plasma, serum, urine, digesta, and feces, following dietary interventions. These findings can help identify biomarkers of gastrointestinal functionality and beyond, as well as investigate mechanistic interactions between diet, host, microbiome, and metabolites. This review aims to summarize the current literature on nutritional metabolomics in pigs and its use to investigate how different dietary approaches impact the gut health of pigs. Here, we critically assessed and categorized the impact of the main macronutrients—carbohydrates, proteins, and fats—along with feed additives such as amino acids, bile acids, and probiotics, as well as feeding strategies like creep feeding, milk replacer introduction, and time-restricted feeding, on the pig metabolome. Additionally, we discuss the potential modes of action of the key affected metabolites on pig gut health.

## Background

Metabolomics is broadly defined as the comprehensive measurement of the metabolome, i.e., all the low-molecular-weight molecules or metabolites in a biofluid, cell, tissue, organ, or organism [1]. With the capability of high-throughput metabolite profiling, metabolomics has numerous applications. These include disease diagnosis and monitoring, drug discovery, environmental toxicology, exposure assessment, and research on the microbiome to identify the influence of the microbiota on host physiology through the production, modification, or degradation of bioactive metabolites. Additionally, metabolomics can evaluate the impact of dietary interventions on various aspects related to host health, such as digestion, metabolism, and the gut microbiome. In this respect, it can accelerate the discovery of biomarkers related to nutrition, health, and disease, as well as support the development of biomarkers and the advancement of precision nutrition [2].

### Metabolomics approaches

Metabolomics analyses can be categorized into two approaches: untargeted and targeted metabolomics. Untargeted metabolomics is a discovery-based method that enables global detection and relative quantification of the metabolome [3]. One major advantage of untargeted metabolomics is data collection without pre-existing hypotheses, which allows uncovering of novel patterns or insights within entire metabolic pathways. However, this is accompanied by the caveat that sample preparation and analytical methods directly affect the coverage of metabolite classes, thus ultimately affecting the assessment of the tested strategy’s impact [4]. Another major challenge in untargeted metabolomics is the identification of the metabolites [5]. Even though the metabolite libraries have expanded considerably during the past years [6], many metabolites still cannot be annotated. On the other hand, targeted metabolomics focuses on measuring well-defined groups of chemically characterized and biologically annotated metabolites, allowing quantitation [7], the latter being an advantage compared to untargeted metabolomics. However, targeted metabolomics relies on a limited metabolome coverage, which increases the risk of overlooking relevant metabolic responses [8].

The main characteristic of metabolomics, compared to other omics fields such as proteomics, transcriptomics, or genomics, is that the metabolome represents the end products of biochemical pathways. As a result, metabolomics is downstream of other omics data and provides a closer reflection of the phenotype of a cell, tissue, or organism [9].

### Nutritional metabolomics

Nutritional metabolomics is the systematic analysis of metabolites in biological fluids or tissues to explore the effects of nutrition on metabolic pathways [10] and has been used to discover biomarkers of nutritional exposure, nutritional status, and nutritional impacts on disease, including gut health [11, 12]. In the area of human research, nutritional metabolomics focuses on those active metabolites, nutrients (or non-nutrients) among the thousands of components in the metabolome that are associated with the effects that different diets have on us [13]. However, similar nutritional metabolomics applications in livestock research are relatively less adopted. Studies have introduced different nutritional approaches to enhance the intestinal health of pigs and to increase the sustainability of production, e.g., by improving feed efficiency and reducing antibiotic use. These approaches include changes in feed ingredients or their levels, e.g., carbohydrate types/sources, protein level and source, and fat composition, as well as feed additives, e.g., essential amino acids, probiotics, prebiotics, antimicrobial peptides, organic acids, and plant extracts [14, 15]. Feed ingredients and additives provide not only necessary nutrients to the animal but also substrates for the mutualistic gut microbiota, which can shape the gut microbiome and metabolome, and thereby contribute to intestinal homeostasis [16].

### Diet-metabolites-gut health

It has proven difficult to define what gut health is and, consequently, how to measure it. According to Kogut and Arsenault [17], the term gut health encompasses a complex interplay between physiological, physical, and immune components that harmonize to support animals to perform at their maximum genetic capacity and cope with internal and external stressors. An optimal gut condition relies on the interaction between the nutrients present in the feed, the gut microbiome, and the intestinal barrier, which facilitates the digestion, absorption, and metabolism of nutrients, thereby allowing for optimum growth and achieving the genetic potential of animals [18]. The gut-derived metabolites, closely linked to energy and nutrient metabolism, are small molecules originating from the digestion of feed and endogenous substrates by animal enzymes or from microbial metabolism. These metabolites are the intermediates or end products involved in numerous metabolic processes, which contribute to the function of the gut and the growth of animals.

The main end products of animal-derived enzyme digestion are amino acids, vitamins, fatty acids, and sugars, which can determine animal growth, development, reproduction, and the gut microbiome [19]. Other compounds derived from the feed, such as phenolic compounds, terpenoids, and alkaloids, have been proven to play an important role in the gut health of pigs. Phenolic compounds, such as benzoic acid and cinnamic derivatives, as well as lignans, are important from a nutritional health perspective [20]. They can either be absorbed in the small intestine or degraded by the microbiota in the large intestine into a range of metabolites. These metabolites can then be absorbed by the host, enter the peripheral blood circulation, or interact locally with the epithelial cells lining the gastrointestinal tract [21].

The gut microbiome-derived metabolites play an important role in determining the gut health of pigs. Metabolites such as short-chain fatty acids, secondary bile acids, and amino acids have beneficial functions and preserve gut homeostasis by, e.g., strengthening the gut-blood barrier, modulating inflammatory responses, and even affecting neurologic functions through the gut-brain axis [22]. However, some potentially toxic microbial-fermentation metabolites, such as ammonia, certain amines (histamine, tyramine, diamines), and phenolic compounds such as phenol, cresol, indole, and skatole from aromatic amino acids produced by the pathobionts in gut microbiome may at high concentrations, impair intestinal epithelial integrity and promote inflammatory reactions, ultimately causing the dysbiosis of the gut [23].

The metabolome is influenced by nutrition, the health status of the host, and the gut microbiota composition. Applying nutritional metabolomics, in combination with other omics, can increase our understanding of the host-microbiome-immune crosstalk in the gut and, thereby, the effect of dietary interventions on pig gut health. Given the increasing number of studies in this area of research, this review aims to summarize the current findings in metabolomics, with a particular focus on untargeted metabolomics approaches, to assess the gut health condition of pigs as affected by dietary approaches.

## Search strategies

**Fig. 1:**
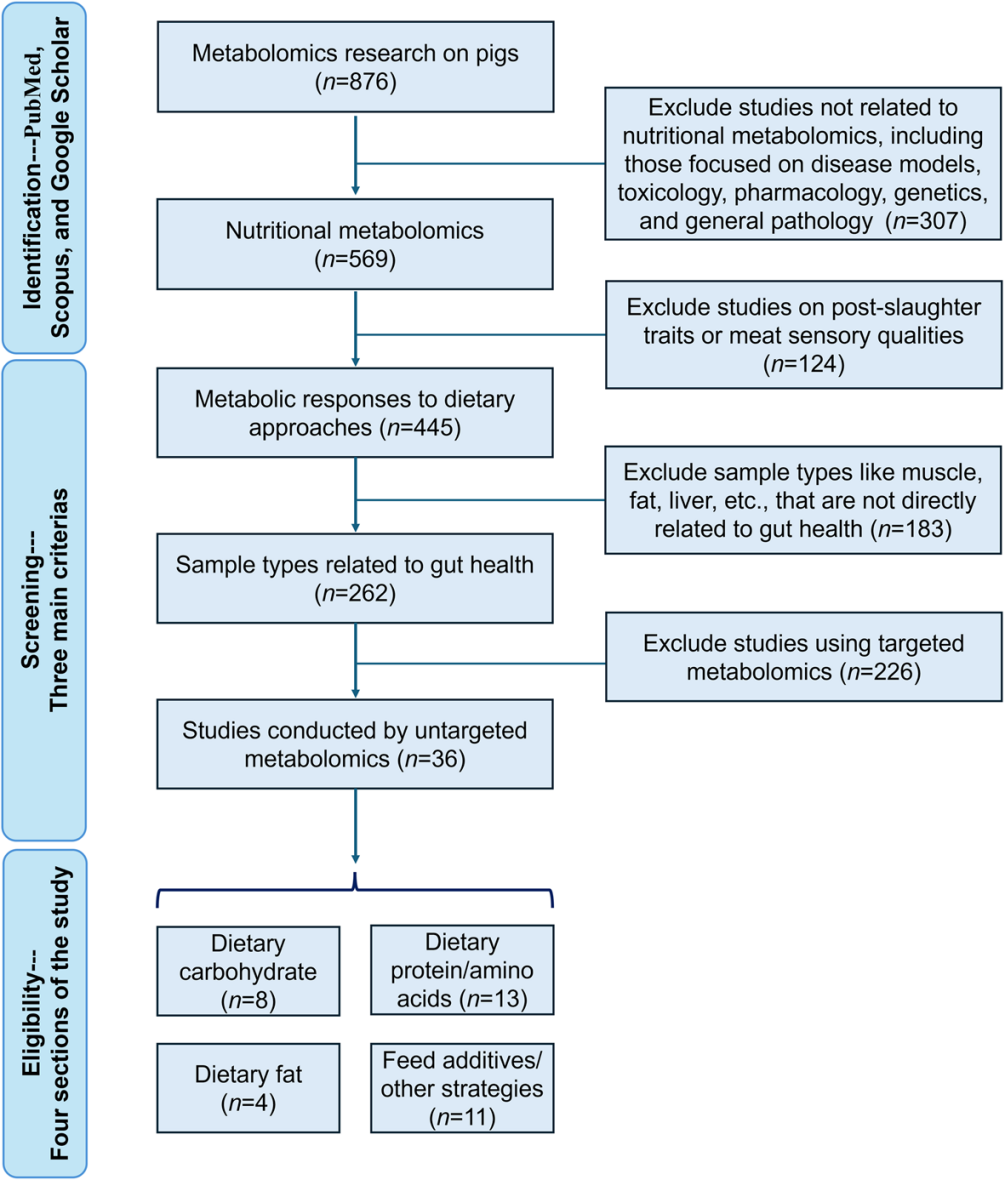
Workflow of literature identification, screening, and inclusion

This review focuses on the impact of the main feeding strategies, e.g., sources and levels of carbohydrates, protein, and fat, and feed additives, e.g., amino acids, bile acids, and probiotics, in relation to pig gut health. A combination of keywords was selected to identify the associated metabolomics studies. These keywords were categorized into three main groups to enhance the search: 1) nutritional approaches (such as dietary fiber/protein level adjustment and dietary probiotics/prebiotics supplementation, 2) sample types (such as ileal/colonic digesta and feces), and 3) metabolomics method information, including metabolomics platforms (LC–MS, GC–MS, NMR, etc.), metabolomics approaches, and terms related to "metabolomics", such as metabolome and metabolite. The workflow, outlining the search strategy used to categorize and filter studies from the databases, is presented in Fig. 1. The review mainly addresses the commonly altered metabolites from the collected papers within the same category of nutritional approach. We included 36 papers to critically assess and discuss the metabolome from sample types such as digesta, feces, urine, and blood. Moreover, the pool of metabolites was further filtered to highlight those proven to be important and related to gut health in pigs or other mammalian species in 42 papers, which could serve as reference markers for evaluating the gut health of pigs under different nutritional approaches.

## Dietary components and metabolome

### Dietary carbohydrates

Carbohydrates from cereal grains comprise the major fraction of pigs’ diets. Carbohydrates are classified into four types based on the degree of polymerization: monosaccharides, disaccharides, oligosaccharides, and polysaccharides. Digestible carbohydrates, such as starch, are those that pigs can digest through the secretion of endogenous enzymes [24]. Starch is the main dietary energy source for pigs, making up ~ 40%−55% of the pig diet on a dry matter basis [25]. Non-digestible carbohydrates, also known as dietary fiber, are anaerobically fermented mainly in the hindgut by the gut microbiota, resulting in metabolites that the pig can absorb and metabolize, stimulating intestinal homeostasis [26]. The amount and source of dietary fiber can have different effects on pig gut function, such as improving carbohydrate and lipid metabolism. Soluble dietary fibers can influence gastrointestinal processes by forming gels that slow gastric emptying, leading to a more gradual absorption of nutrients, which is beneficial for moderating glucose levels and improving carbohydrate metabolism [27]. In contrast, insoluble fibers are not digested and, depending on their structure, can pass through the gastrointestinal tract mostly intact. They add bulk to the stool and enhance gut motility, which helps prevent constipation and maintain regular bowel movements. Moreover, dietary fiber can also lead to changes in the intestinal microbiome and the development of the gut [28]. In particular, certain dietary fiber sources may reduce the risk of postweaning diarrhea and improve the intestinal health of piglets by modulating the gut microbiota and its derived metabolites to provide enough fuel for the colonocytes [29].

Short-chain fatty acids (SCFAs) are derived from microbial fermentation of dietary fiber and play an essential role in regulating gut barrier function, microbiota composition, and immune balance [30, 31]. Much research has been carried out investigating the effect of the level and source of dietary fiber on the concentration of SCFAs, mainly butyrate, propionate, and acetate, in relation to the gut health of pigs. The focus of the current review is specifically on the application of untargeted metabolomics. Therefore, studies focusing only on the fecal profiles of SCFAs will not be included here.

In growing pigs aged 2 to 4 months, metabolomics studies were conducted to elucidate the impact of dietary fiber, including insoluble fiber, soluble fiber, and resistant starch, on gut health, with experimental durations ranging from 3 weeks to 3 months (Table 1). Comparing feeding of high dietary fiber content to low dietary fiber content has shown an increase in the levels of stearic acid in cecum and colon [32, 33], dodecanoic acid in cecum and plasma [34, 35], azelaic acid in both plasma and cecum [35, 36], and 9-hydroxy-10,12-octadecadienoic acid (9-HODE) in cecum [33, 36] (Table 1). These metabolites are associated with fatty acid biosynthesis and linoleic acid metabolism. Specifically, increasing the level of insoluble fiber from 9% to 18% by adding soybean hulls to replace corn and soybean has shown an increase in the levels of O-succinyl-L-homoserine (OSH), associated with cysteine and methionine metabolism in the colon [32]. Increased levels of arachidonic acid, associated with linoleic acid metabolism, and hypoxanthine, associated with purine metabolism, were found when increasing the dietary fiber levels by replacing corn starch in the diet with raw potato starch, which has higher resistant starch content [33].

**Table 1:** The impact of dietary carbohydrates on the metabolome of growing pigs

Strategy	Treatments	Duration	Sample	Key results	Affected pathways	References
Fiber level	9%, 13.5%, 18%, 22.5% NDF by soybean hull 2-month growing pigs	30 d	Colonic content	13.5%, 18% > 9%, 22.5%: oleamide, pentadecanoic acid, palmitic acid, stearic acid 22.5% > 9%, 13.5%, 18%: glycoursodeoxycholic acid, deoxycholic acid, and taurine 13.5%, 18% > 9%: L-serine, O-succinylhomoserine, O-succinyl-L-homoserine	Salivary secretion, phenylalanine metabolism, cysteine and methionine metabolism, and tryptophan metabolism	[32]
Fiber level	Raw potato starch (HDF) vs. corn starch (LDF) 70-day growing pigs	100 d	Cecal and colonic content	Cecum: HDF > LDF: glucose-6-phosphate, maltose, 3-glycerophosphate, fructose, arachidonic acid, 9,12-octadecadienoic acid, oleic acid, hexadecanoic acid, stearic acid, tryptophan, glutamic acid, hypoxanthine, myo-inositol-2-phosphate, and phosphate HDF < LDF: hydrocinnamic acid, nonanoic acid, glyceric acid, alpha-aminobutyric acid, leucine, glycine, aspartic acid, isoleucine and 3-hydroxypyridine Colon: HDF > LDF: maltose, glucose-6-phosphate, fructose, and tryptophan HDF < LDF: alpha-aminobutyric acid, proline, putrescine, phenylalanine, and glycine	Starch and sucrose metabolism, protein synthesis, lipid metabolism, sugar fermentation solution, pentose phosphate pathway, inositol phosphate metabolism, and nucleotide metabolism	[33]
Fiber level	2.4%, 4.2%, 5.5%, 6.8% insoluble fiber by broad bean straw 3-month growing pigs	90 d	Cecal content	Decreased with DF levels: chenodeoxycholate, L-glutamate, and L-pyroglutamic acid Increased with DF levels: 2-oxoadipic acid, caprylic acid, medicagenic acid, sn-glycerol 3-phosphoethanolamine, mannose 6-phosphate, D-galacturonic acid, undecanoic acid, dodecanoic acid, arachidic acid, and succinate	Phosphotransferase system, amino acid metabolism, primary bile acid biosynthesis, secondary bile acid biosynthesis, and fatty acid biosynthesis	[34]
Fiber level	Inulin (LDF) vs. cellulose (HDF) 2-month growing pigs	60 d	Plasma	HDF > LDF: 5-oxoproline, azelaic acid and dodecanoic acid HDF < LDF: hydroxyproline	Arginine and proline metabolism, galactose metabolism, and linoleic acid metabolism	[35]
Fiber level	White wheat flour (LDF) vs. whole grain rye flakes (HDF) 3-month growing pigs	3 weeks	Cecal and colonic content	HDF > LDF: 9,10,13-TriHOME, 9,12,13-TriHOME, 9-HODE, 13-HODE, suberic acid, azelaic acid, sebacic acid, hydroxysebacic acid	Linoleic acid metabolism and fatty acid metabolism	[36]
Fiber source	Rye bread (RB) vs. wheat bread (WB) 4-month growing pigs	3 weeks	Plasma urine	Plasma: RB > WB: arachidonic acid derived oxylipins, eicosanoids such as 5-hydroxy-6,8,11,14-eicosatetraenoic acid and 5–12-dihydroxy-6,8–10,14-eicosatetraenoic acid RB < WB: linoleic acid derived oxylipins such as an isomeric mixture of 9-HODE and 13-HODE Urine: RB > WB: enterolactone, hippuric acid, 2-methylhippuric acid, ferulic acid, p-cresol, 3-phenyllactic acid, 5-methoxy-3-indoleacetic acid, N-feruloylglycine, quinol and homovanillic acid	Arachidonic acid metabolism, linoleic acid metabolism, Phenolic acids metabolism/fermentation, Tyrosine and 4-hydroxyphenylacetic acid metabolism/fermentation	[46]
Starch source	Pea starch vs. tapioca starch vs. corn starch 4-month growing pigs	40 d	Colonic content	Pea starch > tapioca starch: galactose, fucose, N-acetylgalactosamine, glycerate, stearic acid, capric acid, linoleic acid, lactate, uracil, and pantothenic acid Pea starch < tapioca starch: leucine, glycine, putrescine, tyramine, indole-3-acetic acid, p-cresol, and hydroxylamine Corn starch < tapioca starch: glycine, cholesterol, and hydroxylamine Corn starch > tapioca starch: fucose, glucose, ribose, N-acetylgalactosamine, glycerate, stearic acid, and capric acid	Carbohydrate metabolism and amino acid metabolism	[49]
Starch source	Pea starch vs. tapioca starch 4-month growing pigs	44 d	Cecal content	Pea starch > tapioca starch: valine, tryptophan, isoleucine, γ-aminobutyric acid, dimethylglycine, indolelactic acid, 3-indolepropionic acid, N-acetylserotonin, pyroglutamic acid, N-acetyltryptophan, monoacylglycerol, tetradecanoylcarnitine, taurocholic acid, 12,13-DiHOME, and ricinoleic acid Pea starch < tapioca starch: histamine, indole, taurochenodeoxycholic acid, dodecanedioic acid, and octadecatrienoic acid	Biosynthesis of amino acids, carbon metabolism, amino sugar and nucleotide sugar metabolism, starch and sucrose metabolism, and arginine and proline metabolism	[50]

*NDF* Neutral detergent fiber, *HDF* High dietary fiber, *LDF* Low dietary fiber, *9,10,13-TriHOME* 9,10,13-Trihydroxyoctadecatrienoic acid, *9-HODE* 9-Hydroxyoctadecadienoic acid, *12,13-DiHOME* 12,13-Dihydroxyoctadecadienoic acid. Symbols ' > ' and ' < ' indicate relative comparisons in metabolite concentrations between dietary treatments. For example, 'diet A > diet B' means that the concentration of a given metabolite in samples after feeding with diet A is higher than after feeding with diet B, while 'diet A < diet B' indicates a lower concentration following diet A compared to diet B

Changes in the metabolome by increasing the levels of dietary fiber, as reviewed here, can contribute to better gut health. Dietary stearic acid and dodecanoic acid have been shown to improve gut morphology and epithelial cell turnover in weaned piglets [37]. Additionally, an increased level of dodecanoic acid in the digesta may be related to the higher level of soybean oil in the high dietary fiber diet. Azelaic acid has been shown to increase the secretion of glucagon-like peptide-1 and regulate intestinal inflammation [38]. Oxidation products of linoleic acids, such as 9-HODE and 13-HODE, have been reported to regulate macrophage differentiation and pro-inflammatory action, which can regulate cellular pathways through interaction with cell surface receptors during tissue inflammation [39]. Studies have shown that 9-HODE acts pro-inflammatory, while 13-HODE exhibits anti-inflammatory functions [40, 41]. Concerning amino acid metabolism, OSH is a crucial precursor of L-methionine biosynthesis by gut microbiota [42, 43]. L-methionine production can provide methyl groups for many critical physiological processes, resulting in higher antioxidant status, improved morphology, and barrier function of the gut epithelium of piglets [44, 45]. A recent study has, however, shown that too high levels of insoluble dietary fiber, e.g., soybean hulls, may negatively affect the gut health of growing pigs. It was demonstrated that feed containing 22.5% compared to 13.5% and 18% insoluble fiber led to a decrease in intestinal microbial diversity with loss of bacteria associated with gut homeostasis [32]. Additionally, the same study found that the morphology and development of the duodenum and ileum, along with average daily gain, were adversely affected. These effects may be attributed to the high insoluble fiber content, which accelerates the passage of chyme through these intestinal segments, thereby reducing nutrient digestibility and absorption. Lastly, the amount of stearic acid in the colonic content decreased, which could affect the turnover of intestinal epithelial cells, as mentioned in the previous section. These factors may reduce the intestinal defense against pathogens.

Nørskov et al. [46] compared the plasma metabolome when feeding a diet based on whole grain rye meal and rye bran or a diet based on refined wheat flour with dietary fiber adjusted to similar levels and found that different fiber sources had various impacts on the metabolite composition in the plasma of growing pigs. In the plasma metabolic profiles, arachidonic acid-derived oxylipins were higher when feeding the rye diet. In contrast, linoleic acid-derived oxylipins such as 9-HODE and 13-HODE were found at a lower concentration when feeding rye compared to wheat as the dietary fiber source [46]. However, more studies are needed to elucidate the metabolome in the gut environment to find the most suitable dietary fiber source for different ages of pigs.

Dietary starch is the major energy source in pig feed. Different dietary starch sources with distinct molecular structures can affect the rate and degree of their enzymatic hydrolysis in the small intestine, resulting in various amounts of starch accessible to microbial fermentation in the hindgut [47, 48]. Studies have shown that feeding pigs with pea starch or tapioca starch, which have different amylose and amylopectin ratios, affected the metabolite composition in both colonic [49] and cecal [50] content differently. Compared to tapioca starch, pigs fed with pea starch had higher levels of some amino acids and metabolites associated with amino acid metabolism (valine, tryptophan, isoleucine, and γ-aminobutyric acid, dimethylglycine, indoleacetic acid, N-acetylserotonin, and 3-indolepropionic acid) [50]; carbohydrate metabolism (galactose and fucose) [49]; and fatty acid metabolism (monoacylglycerol, tetradecanoylcarnitine, and taurocholic acid) [50]. At the same time, higher levels of p-cresol, tyramine, and putrescine [49], but lower levels of histamine and indole [50], which are involved in amino acid metabolism, were detected. Stearic acid and decanoic acid (capric acid) in colon, and taurochenodeoxycholic acid, prostaglandin D1, and dodecanedioic acid in cecum, which are involved in fatty acid metabolism, [49, 50] were found at lower levels when feeding pigs with pea starch compared to tapioca starch. Capric acid can protect against inflammation, inhibit antioxidant activity, stimulate neurotransmitters, act as an antimicrobial agent, and enhance the barrier function in pig gut [51, 52]. A lower histamine level was observed in the colonic contents of pigs fed pea starch compared to those fed tapioca starch. This may benefit gut health because, at high levels, histamine can induce local inflammation, with a risk of disturbing the mucosal immune homeostasis [53].

In summary, both the level and source of dietary fiber and starch influence the gut metabolome in pigs, which was reported to impact gut health. A higher dietary fiber intake, particularly with a greater proportion of insoluble fiber, stimulates linoleic acid metabolism and supports the immune system by modulating oxylipins. Additionally, it can enhance fatty acid metabolism by regulating stearic acid, azelaic acid, and dodecanoic acid, which are associated with the stimulation of intestinal barrier function. Furthermore, dietary starch sources can influence the digesta levels of indole and p-cresol, as well as biogenic amines, including histamine, tyramine, and putrescine, making it a potential nutritional strategy for maintaining gut homeostasis.

### Dietary protein and supplementary amino acids

The quantity and quality of dietary protein can influence animal feed intake and metabolism, consequently impacting gut health and growth [54]. Metabolomic profiles in blood, gut content, and feces have been studied to evaluate the impact of different dietary protein levels or protein sources on pig gut health [55–59]. Piglets, especially weaners, have a low capacity to digest protein, which increases the amount of undigested protein in the large intestine, thereby increasing the amount of available fermentable protein [60]. Excess fermentable dietary protein can shift the microbiota composition and change the levels of specific metabolites [23, 61]. High levels of metabolites such as polyamines, biogenic amines, and ammonia, derived from microbial proteolytic activity in the gut, may have detrimental effects on the host, including impairment of the intestinal epithelial integrity, promotion of inflammatory reactions, increased secretion of gastric acid, and alteration of mucosal ion secretion, leading to an increased risk of post-weaning diarrhea (PWD) [62].

Results from studies investigating the impact of dietary protein on pig gut health are summarized in Table 2. Studies involving piglets aged 14 to 35 d, fed diets with either low protein content (16%−18%) or high protein content (20%−30%) for an experimental period of 14–28 d, have shown an increase in the colonic levels of tryptophan, and the metabolites associated with tryptophan metabolism, serotonin, 3-indoleacrylic acid, and 3-indoleacetic acid [55, 56], whereas a decreased level of tryptophan was found in serum [57] (Table 2). Furthermore, stimulation of vitamin metabolism was found when feeding piglets with low dietary crude protein (16%−18%) [55, 57]. Increased levels of vitamin and vitamin-derivatives such as menaquinone, nicotinate, and dihydrofolate were found in colon [55] as well as dehydroascorbic acid were measured in serum in pigs fed a low protein diet [57]. Fatty acid derivatives such as 9-HODE and azelaic acid have also been shown to increase in the colon when feeding low-protein diets [56]. High valeric acid, associated with gut microbiota metabolism [56], and a decrease in the levels of arachidonoylmorpholine, involved in arachidonic acid metabolism [56], and histamine and 3-N-methyl-L-histidine, involved in histidine metabolism [55], have been measured in the colon of pigs fed low protein diets as compared to high protein diets. These metabolites all play essential roles in aspects affecting gut health. Serotonin and 3-indoleacrylic acid, produced by the gut microbiota through tryptophan hydrolyzation and decarboxylation, benefit the host’s immunity and metabolic homeostasis as well as promote intestinal barrier function by stimulating goblet cell differentiation and mucosal immune cell production [63, 64]. Some results indicate that dehydroascorbic acid, an oxidized form of ascorbic acid, could be a dietary vitamin C source in pigs [65]. Vitamin C has potent antioxidant, immunomodulatory, and anti-infectious effects in the gut [66, 67]. Valeric acid, originating from plant-based components of the diet, has been found to influence gut microbiome composition and increase the expression of pro-inflammatory cytokine. These effects contribute to its strong protective role in gut injury by enhancing intestinal epithelial integrity [68]. Furthermore, a decreased level of arachidonoylmorpholine could be related to improved pig gut health and reduced risk of PWD. Studies have shown that increased arachidonoylmorpholine, caused by gut microbiota dysfunction, may induce disordered arachidonic acid metabolism, further leading to dysfunction in maintaining intestinal epithelial homeostasis [69].

**Table 2:** The impact of dietary crude protein and supplementary amino acids on the metabolome of growing pigs

Strategy	Treatments	Duration	Sample	Key results	Affected pathways	References
Crude protein level	16% vs. 20% crude protein with the adjustment of soybean meal 21-day-old weaned piglets	14 d	Colonic content	HP > LP: histamine, 3-N-methyl-L-histidine HP < LP: D-tryptophan, serotonin, 3-indoleacrylic acid, dihydrofolate, menaquinone, nicotinate	Tryptophan metabolism, vitamin metabolism,histidine metabolism, and glycerophospholipid metabolism	[55]
Casein protein level	17% vs. 30% casein protein 28-day-old weaned piglets	14 d	Ileal and colonic content	HP > LP: (2-oxo-2,3-dihydro-1H-indol-3-yl)acetic acid, cucurbitacin I, L-hydroxyisovaleric acid, orotate, pentanoate, pentanoic acid, 2-methyl-4,5-benzoxazole, 2-methyl-4,5-benzoxazole, 5-ethyl-5-(pentan-2-yl)−1-((2S,3R,4S,5S,6R)-3,4,5-trihydroxy-6-(hydroxymethyl)tetrahydro-2H-pyran-2-yl)pyrimidine 2,4,6(1H,3H,5H)-trione, damascenine, Trp-Pro-Ser, Ala-Ala-Glu, 3,6-dimethoxyestra-1,3,5(10),6,8-pentaene-17 beta-carboxylic acid methyl ester, eremophilenolide, D-erythro-Sphingosine C-20, PE(16: 0/0: 0), LysoPC(14: 0), lucanthone, N-methyl-1H-indole-3-propanamide, methyldopa, 3-hydroxy-5-chola-8(14), 11-dien-24-oic acid, oseltamivir, retinol, 2-(4-methyl-5-thiazolyl)ethyl decanoate, mupirocin, hericenone H, cadiamine, leiokinine A, aklomide, Met-Gly-Pro-Thr, 2(N)-methyl-norsalsolinol, arachidonoylmorpholine, and 5-androstan-3-ol-17-one sulfate HP < LP: Ile Pro, 19-norandrosterone, 9-HODE, 9-HOTrE, azelaic acid, beta-hydroxymyristic acid, glycocholate, N-Ac-Tyr-Val-Ala-Asp-CHO, sebacic acid, taurocholic acid, creatine, goniothalesdiol, 14,15-LTA4, tephrowatsin B, Dimefuron, PGA1, 3-(2-methylpropanoyloxy)−8-(3-methylbutanoyloxy)-9,10-epoxy-p-mentha-1,3,5-triene, prostaglandin I2, 3-indoleacetic acid, 5-propylideneisolongifolane, 11-dehydro-TXB3, Kamahine C, 3-(2,4-cyclopentadien-1-ylidene)-5alpha-androstan-17beta-ol,(9R,13R)-12-oxophytodienoic acid, stearidonic acid, 5-methoxyhydnocarpin-D, Trp-Glu-Glu, Asn-Arg-Lys-Ala, and 6-[3]-ladderane-1-hexanol	Bile acid biosynthesis, alpha-Linolenic acid metabolism, phospholipid biosynthesis, arachidonic acid metabolism, fatty acid biosynthesis, retinol metabolism, arginine and proline metabolism, pyrimidine metabolism, tryptophan metabolism, and glycine and serine metabolism	[56]
Crude protein level	12%, 18%, 24% crude protein with the adjustment of soybean meal 35-day-old piglets, weaned at 14 days of age	28 d	Serum	24% > 12%: indole-3-acetate, lactobionic acid, aconitic acid, oxoproline, valine, tyrosine, 2-ketoisocaproic acid, pyrrole-2-carboxylic acid 24% < 12%: N-acetylmannosamine, glucose-1-phosphate, aminomalonate, putrescine 24%.18% > 12%: 4-hydroxyhippuric acid, arabitol, pinitol, hexitol, xylitol, conduritol-beta-epoxide, saccharic acid, UDP-glucuronic acid, isoleucine, tryptophan, glycine, isohexonic acid, *trans*-4-hydroxy-L-proline, N-acetylaspartic acid 24%.18% < 12%: 2-hydroxyglutaric acid, glucose, hydroxycarbamate, glutamic acid, phenylethylamine, cholesterol 18% > 12%.24%: dehydroascorbic acid	Carbohydrate metabolism, amino acid metabolism, fatty acid metabolism, and vitamin metabolism	[57]
Crude protein level	13% vs. 17% crude protein with the adjustment of corn-soybean meal 120-day-old 4-month growing pigs	51 d	Colonic content	LP compared to HP, a total of 156 differentially abundant metabolites in LC–MS (ESI-) were identified, including 32 increased and 124 decreased abundant metabolites. Similarly, 278 metabolites in LC–MS (ESI+) were identified, including 126 increased and 152 decreased abundant metabolites	Phosphotransferase system, ascorbate and aldarate metabolism, the HIF-1 signaling pathway, and asthma, glutathione metabolism, inflammatory mediator regulation of TRP channels, the Fc epsilon RI signaling pathway, linoleic acid metabolism, degradation of aromatic compounds, biosynthesis of alkaloids, ascorbate and aldarate metabolism, the HIF-1 signaling, and glutathione metabolism	[58]
Crude protein level	15% vs. 13% crude protein with the adjustment of corn-soybean meal 70-day-old growing pigs	100 d	Colonic content	HP > LP: benzoate, lyxose, and memy HP < LP: xanthine, hypoxanthine, stearic acid, glyceric acid, methyl phosphate, malic acid, leucine, glutamic acid, and tyrosine	Phenylalanine, glycine, serine, alanine, aspartate, glutamate, purine, threonine metabolism, and pyruvate metabolism	[59]
Branched-chain amino acid supplementation	0.82% Leucine, 0.55% isoleucine, and 0.57% valine supplementation in the diet 28-day-old weaned piglets	4 weeks	Plasma	LP < LP + BCAA: uridine, isoleucine, valine, glycine, oxoproline, 2-hydroxyvaleric acid, 3-hydroxybutyric acid, 2-ketoisocaproic acid, aminomalonate, indole-3-acetate, and indole-3-lactate LP > LP + BCAA: 5-aminovaleric acid, ribonic acid, arabitol, 1,5-anydroglucitol, urea, threonine, methionine, and 2,3-dihydroxybutanoic acid	Phenylalanine metabolism, fatty acid synthesis, valine, leucine and isoleucine degradation and biosynthesis	[76]
Branched-chain amino acid supplementation	The diets were planned to contain 0.42, 0.46, 0.50, 0.54, 0.58, and 0.62 SID Ile/Lys; 0.58,0.62, 0.66, 0.70, 0.74, and 0.78 SID Val/Lys; and 0.70, 0.80, 0.90,1.00, 1.10, and 1.20 SID Leu/Lys in the Ile, Val, and Leu dose 35-day-old weaned piglets	15 d	Plasma	Increasing SID Ile/Lys: Increased level of 3-methyl-2-oxovaleric acid, tyrosine, hypoxanthine, and indoxylsulfuric acid Decreased level of glycocholic acid, taurocholic acid, lysoPC, tauroursodeoxycholic acid Increasing SID Val/Lys: Decreased level of docosahexaenoic acid ethyl ester, hippuric acid, tryptophan, and arachidonic acid ethyl ester Increasing SID Leu/Lys: Decreased level of phenylalanine, α-ketoisovaleric acid, creatine, and isoleucine	Primary and secondary bile acid biosynthesis, primary and secondary bile acid biosynthesis, purine metabolism, inflammatory mediator regulation of TRP channels, isoleucine degradation, phenylalanine metabolism, valine degradation and biosynthesis, fat metabolism	[77]
Branched-chain amino acid supplementation	Low crude protein diet supplemented with leucine, isoleucine, and valine 21-day-old weaned piglets	5 weeks	Plasma	Increased level of azelaic acid, valine, beta-alanine, α-tocopherol, cystine and norvaline Decreased levels of creatinine, urea, tyrosine, and phenylalanine	Lipid metabolism, amino acid metabolism, vitamin metabolism, creatinine metabolism	[78]
Amino acid supplementation	lysine restriction diet (70% of the control) 4-week-old weaned piglets	16 weeks	Ileal content	Increased level of trigonelline, thiabendazole, p-nitrobenzoic acid, orotidine, N-(2-furoyl) glycine, l-glutamic acid, l-cysteinesulfinic acid, dl-glutamic acid, d-glucuronic acid, 8-hydroxy-2-deoxyguanosine, aminoethylphosphonic acid, (−)-epicatechin, trigonelline, and N-nitrosomorpholine	Glutamine and glutamate metabolism, taurine metabolism, alanine, aspartate and glutamate metabolism, vitamin B_6_ metabolism, pentose and glucuronate metabolism	[81]
Amino acid supplementation	1% L-Gln diet vs. isonitrogenous control diet (with L-Ala) 21-day-old weaned piglets	1 month	Serum	Increased level of dimethylmalonic acid, 2-hydroxybutyric acid, aminomalonic acid, α-methyltyrosine, Pro, octadecanoic acid, and Tyr Decreased level of acetic acid, glycerol, mannonic acid lactone, d-fructose, and d-xylose	Glutamine and glutamate metabolism, taurine metabolism, alanine,aspartate and glutamate metabolism, vitamin B_6_ metabolism, and pentose and glucuronate metabolism	[82]
Amino acid supplementation	0.4% cysteine supplementation Pregnant sows	late pregnancy (85–90 days of gestation)	Plasma	Increased level of 2-pyrrolidinone, hypotaurine, acetylcysteine, and alanyl-histidine Decreased levels of pyroglutamic acid, 3-methylcrotonylglycine, cytosine, (S)-2,3,4,5-tetrahydropyridine-2-carboxylate, guanine, and alanyl-histidine	Amino acid metabolism and glutathione synthesis	[83]
Protein source	5%&10% insect meat supplementation *Tenebrio molitor* 4-week-old weaned piglets	1 month	Plasma	Insect feeding vs. control: Increased level of alanine, citrulline, glutamate, proline, serine, tyrosine and valine	Amino acid metabolism	[87]
Protein source	Diet with 20% coarse rapeseed meal, 4% rapeseed hulls 7-week-old weaned piglets	3 weeks	Digesta samples	Rapeseed feeding vs. control: Increased level of sinapine, sinapic acid, and gluconapin Decreased level of daidzein	Isoflavonoid biosynthesis, degradation of flavonoids, and biosynthesis of secondary metabolites	[89]

*HP* High crude protein, *LP* Low crude protein, *Trp-Pro-Ser* Tryptophan-Proline-Serine, *Ala-Ala-Glu* Alanine-Alanine-Glutamic acid, *PE(16: 0/0: 0)* Phosphatidylethanolamine, *LysoPC(14: 0)* Lysophosphatidylcholine, *N-Ac-Tyr-Val-Ala-Asp-CHO* N-Acetyl-Tyrosyl-Valyl-Alanyl-Aspartal (Aldehyde form), *9-HODE* 9-Hydroxyoctadecadienoic acid, *9-HOTrE* 9-Hydroxyoctadecatrienoic acid, *PGA1* Prostaglandin A1, *14,15-LTA₄* 14,15-Leukotriene A₄, *BCAA* Branched-chain amino acids, *SID Ile/Lys* Standardized ileal digestible isoleucine to lysine. Symbols ' > ' and ' < ' indicate relative comparisons in metabolite concentrations between dietary treatments. For example, 'diet A > diet B' means that the concentration of a given metabolite in samples after feeding with diet A is higher than after feeding with diet B, while 'diet A < diet B' indicates a lower concentration following diet A compared to diet B

Reducing dietary protein levels in pigs aged 2 to 4 months, over a period of 50 to 100 d, has been adopted to evaluate health status and assess whether it can minimize nitrogen loss and thereby improve the sustainability of pig production. Sampling and measurements were conducted during both the growing and finishing stages [70, 71]. These studies have also shown different metabolite profiles in the colonic content of growing pigs when feeding 13% dietary crude protein compared to 15%−17%. These include an increase in the levels of arachidonic acid [58], stearic acid, and hypoxanthine [59], and a decrease in levels of histamine and vitamin C in the low protein-fed animals [58]. Hypoxanthine in colonic content has been shown to modulate the colonic epithelial energy metabolism and improve the barrier function [72]. A decreased hypoxanthine level in the gut has been linked to increased microbial utilization and breakdown due to gut microbiome dysbiosis, as well as supporting colonocyte energy through the purine salvage pathway for mucosal repair [73].

Supplemental crystalline amino acids are essential for pig production, as they reduce the use of protein supplements while maintaining the ideal protein profile in the diets. Feeding pigs with low crude protein diets supplemented with individual amino acids is a dietary strategy to reduce N excretion and the risk of PWD without impairing growth performance [74]. Additionally, supplementing branched-chain amino acids (BCAAs), which play a role in energy homeostasis, muscle protein synthesis, and immune function, in low crude protein diets has been reported to support intestinal function and maintain the growth performance of weaned piglets [75]. Valine, leucine, and isoleucine supplementation in very low-protein or protein-restricted diets to compensate for energy requirements has been studied and shown to induce significant changes in plasma metabolic profiles in weaned piglets aged 3 to 5 weeks over an experimental period of 2 to 5 weeks [76–78] (Table 2). BCAA supplementation resulted in increased levels of valine, glycine, tyrosine, hypoxanthine, indole-3-acetate, and indole-3-lactate [76–78]; decreased levels of glycocholic acid and taurocholic acid [77] and creatine and creatinine [77, 78] in plasma metabolite profiles. Recent evidence has shown that indole-3-acetate, an important microbial tryptophan metabolite, can modulate intestinal homeostasis and suppress inflammatory responses [79]. One study has also revealed that lower blood creatinine concentration correlates with decreased colonic inflammation and improved expression of colon tight junction proteins [80]. Other studies have also used metabolomics profiling to evaluate the impact of restricting the essential amino acid lysine or supplementing nonessential amino acids, such as L-glutamine and cysteine, in feed on pig health [81–83]. In late-pregnancy sows at 85 to 90 days gestation, an increase of hypotaurine and acetylcysteine, involved in glutathione biosynthesis, has been found in the serum metabolome under 0.4% cysteine supplementation in the diet [83]. Acetylcysteine in the gut can reduce inflammation, alleviate oxidative stress, improve energy status, and attenuate tissue damage in the intestine of lipopolysaccharide-challenged piglets [84]. Moreover, the effects of acetylcysteine are associated with cell signaling and tight junction signaling pathways [85].

Besides the supplementation of amino acids to the feed, different protein sources, such as insects, rapeseed, and algae, can also provide different amino acid compositions. They can impact gut barrier function, microbiota composition, and inflammatory responses by differently derived metabolites. In recent years, insects have been investigated as a sustainable alternative protein source for pigs. Insects are easy to grow and have a balanced nutritional value [86]. Meyer et al. [87] have shown that supplementing 5%−10% *Tenebrio molitor* insect meal at the expense of soybean meal in the feed of piglets over a 1-month feeding period can increase the amino acids alanine, citrulline, glutamate, proline, serine, tyrosine, and valine concentration in plasma metabolome as well as the amino acid metabolite methionine sulfoxide. Methionine sulfoxide might have the potential to induce macrophage polarization and modulate oxidative stress in the gut [88]. Regarding different plant-based protein sources, rapeseed is another abundant and inexpensive protein source, an alternative to soybean meal, and has been shown to increase the levels of sinapine, sinapic acid, and gluconapin in small- and large intestinal samples [89]. Sinapine has been reported to influence the gut microbiota composition by decreasing the ratio of Firmicutes to Bacteroidetes and increasing the abundance of beneficial microbes [90]. Another study investigated the impact of supplementing the diet with 10% of a microalgae, Spirulina, found no significant differences in the ileal metabolomic profiles [91].

In summary, the presented studies show that reducing dietary crude protein levels from 20%−30% to 16%−18% for weaned piglets and from 15%−17% to 13% for growing pigs can influence arachidonic acid metabolism in the gut, which is associated with intestinal epithelial homeostasis and a reduced risk of diarrhea. Reducing crude protein also led to a higher hypoxanthine level in the digesta, which could indicate improved barrier function and microbiome homeostasis. Additionally, reducing dietary crude protein increased azelaic acid and 9-HODE in the gut, which can enhance gut barrier function and gut immunity status, respectively. BCAA and specific amino acid supplementation can modulate intestinal homeostasis by suppressing inflammatory responses and alleviating oxidative stress. Based on the metabolomics data, plant-based protein sources like rapeseed meal have beneficial effects on gut health as they can introduce phytochemical metabolites that reduce harmful microbiota and maintain gut homeostasis. However, summarizing the effects of plant-based protein sources is challenging, and more studies are needed to examine their effectiveness.

### Dietary fat

Fat is an essential macronutrient for pigs. An increasing level of fat in the diet can decelerate the passage of feed through the digestive tract, allowing more time for better digestion and absorption of other nutrients [92]. For example, this mode of action has been proposed for the higher digestibility of amino acids observed with increasing levels of dietary fat in pigs [93, 94]. On the other hand, the digestibility of fat decreases when the level of dietary fiber increases, possibly due to the physical binding of the fiber to fat and altered gut transit time [95]. Soluble fibers can form gels or viscous solutions in the gastrointestinal tract, which may encapsulate fat molecules. Moreover, insoluble fibers can interact with fat through physical entrapment during digestion. Various fat and oil sources are available in feed production, including animal fats, plant oils, hydrogenated fats, and free fatty acids [96]. The levels and sources of dietary fat are important for pigs because they can affect lipid metabolism and body composition [97].

Metabolomics has been adopted to elucidate the impact of different dietary fat levels and sources on the metabolism of pigs by examining the metabolite composition in blood, gut content, and liver tissue [98–100] (Table 3). However, the aim of these studies has been mainly to use pigs as a model for human diseases. Therefore, the type and amount of dietary fat used in these studies may not be directly applicable to diets for growing pigs. The pig breed used in these studies was the Ossabaw miniature pig, which has physiological and anatomical similarities to humans regarding the cardiovascular system and has been established as a model for studying metabolic syndrome as well as obesity, inflammatory bowel disease, and cardiovascular diseases [101]. The metabolomics studies conducted on Ossabaw miniature pigs have shown that pigs fed with a high-fat diet (30%−44% of total kcal from fat) compared with a regular diet (8%−11% of total kcal from fat) by adding extra hydrogenated soybean oil have higher levels of chenodeoxycholic acid [98, 99], tauroursodeoxycholic acid [99, 100], glycocholic acid [99, 100], phosphatidylcholine [100], phosphatidylglycerol [99], and glycerophospholipids [99] in a range of sample types, including plasma, colonic content, and liver tissue. The increased metabolites are related to bile acid and lipid metabolism. Some of the metabolites in the gut metabolome under the increase dietary fat might be beneficial for the gut. Chenodeoxycholic acid and tauroursodeoxycholic acid levels in the gut have been shown to maintain intestinal homeostasis and shape innate and systemic immune responses under inflammatory conditions by modulating macrophage metabolism [102, 103]. On the other hand, increased dietary fat can lead to the production of phosphatidylglycerol and glycerophospholipids, which may indicate a risk state in animals [104]. High fat intake can cause dysbiosis and inflammation in the gut and, consequently, an increased concentration of phosphatidylglycerol in the serum, a process regulated by the gut microbiota via endotoxins that can modulate adipose tissue homeostasis in obesity [104]. Gong et al. [105] also found that the increase of colonic glycerophospholipids was associated with abnormal composition of the gut microbiota in a depression model in mice.

**Table 3:** The impact of dietary fat on the metabolome of piglets and growing pigs

Strategy	Treatments	Duration	Sample	Key results	Affected pathways	References
Fat level	18.5% of total kcal from protein, 71% from carbohydrates, and 10.5% from fat vs. 13% of total kcal from protein, 57% from carbohydrates, and 30% from fat 8-week-old growing pigs	24 d	Brain Kidney Muscle	High dietary fat compared to low: Brain: increased S-adenosylhomocysteine and decreased glutathione Kidney: decreased indoxyl sulfate Muscle: increased 7α-hydroxy-3-oxo-4-cholestenoic acid, chenodeoxycholic acid, uridine diphosphate glucuronic acid, and inosine	Purine metabolism, primary bile acid biosynthesis, secondary bile acid biosynthesis, tryptophan metabolism, cysteine and methionine metabolism, Bile secretion, and ABC transporters	[98]
Fat level	21% of total kcal from protein, 68% from carbohydrates, and 11% from fat vs. 16% of total kcal from protein, 40% from carbohydrates, and 44% from fat 4-week-old piglets	11 weeks	Plasma Feces	High dietary fat compared to low: Plasma: increased chenodeoxycholic acid glycine conjugate, tauroursodeoxycholic acid, hyodeoxycholic acid, deoxycholic acid glycine conjugate, and glycocholic acid Feces: increased phosphatidic acid, phosphatidylglycerol, and glycerophospholipids	Primary bile acid biosynthesis, bile acid biosynthesis, and cholesterol metabolism	[99]
Fat level	3.75% vs. 9.12% dietary fat 19-week-old growing pigs	18 weeks	Plasma Liver Urine Proximal colon	High dietary fat compared to low: Plasma: increased glycodeoxycholic acid and glycoursodeoxycholic acid Liver: increased glycocholic acid and trihydroxyoxocholanyl-glycine Urine: decreased dihydroxyindole and salicyluric acid Proximal colon: increased LPC(P-18:0) and decreased LPC (20:2)	Bile acids metabolism, tyrosine metabolism, and glycerophospholipid metabolism	[100]
Fat source	Diets containing 3%, 6%, and 9% oxidized corn oil vs. control diet without oxidized corn oil 19-day-old piglets	35 d	Liver Serum	In liver, 9% oxidized corn oil vs. control: Increased levels of NAD⁺, AMP, and decreased levels of glutathione (GSH), and oxidized glutathione (GSSG) In serum, 9% oxidized corn oil vs. control: Decreased levels of alanine, ascorbic acid, glutamate, carnosine, and tryptophan	Amino acid metabolism (especially tryptophan metabolism), purine metabolism, fatty acid metabolism, and glutathione metabolism,	[108]

*ABC transporters* ATP-binding cassette transporters, *LPC* Lysophospholipids, *NAD* Nicotinamide adenine dinucleotide, *AMP* Adenosine monophosphate

In pig nutrition, lipid sources are incorporated to enhance energy density, supply essential fatty acids, and improve feed quality. However, ingredients rich in polyunsaturated fatty acids, such as vegetable oils and animal by-products, are more susceptible to oxidative rancidity [106]. Excessive intake of oxidized lipids, such as oxidized corn oil, has been shown to impair growth performance, increase oxidative stress in tissues, and negatively affect meat quality in growing-finishing barrows [107]. Metabolomic analyses have revealed that diets containing 9% oxidized corn oil disrupt amino acid homeostasis and glutathione metabolism [108]. Specifically, elevated levels of NAD⁺, AMP, reduced glutathione (GSH), and oxidized glutathione (GSSG) were observed in the liver, while serum levels of alanine, ascorbic acid, glutamate, carnosine, and tryptophan decreased. The reduction in serum tryptophan alongside increased hepatic NAD⁺ suggests activation of the tryptophan-NAD⁺ biosynthesis pathway as a protective response to oxidative stress. This adaptive mechanism may help sustain NAD⁺ levels, essential for antioxidant defenses and gut integrity. If this compensatory mechanism is insufficient or impaired, the accumulation of oxidative stress can overwhelm the body's antioxidant defenses. This may lead to biomolecular damage, mitochondrial dysfunction, epithelial cell injury, and the activation of inflammatory pathways, ultimately compromising gut health [109].

In summary, these studies demonstrate that a high-fat diet can lead to an increase in metabolites involved in bile acid and lipid metabolism. The shift in metabolite composition suggests that high-fat diets may elevate certain bile acids and fatty acids, potentially improving gut health. However, these same diets can also cause a significant increase in lipid and glycerophospholipid metabolism, which may compromise gut function by altering the gut microbiome composition, leading to dysbiosis and local inflammation. Additionally, the source of dietary lipids can influence gut health, particularly when increased oxidative stress arises from the consumption of oxidized fats rich in unsaturated fatty acids. Therefore, careful formulation of pig diets is essential to mitigate metabolic disturbances and maintain optimal gut function.

### Feed additives and other feed strategies

Feed additives have been widely used in the swine feed industry to improve animal growth performance by promoting growth rate, feed conversion, and overall health status. These additives can contribute to pig gut health by preventing the tissue-damaging inflammatory response to pathogens, toxins, and stress factors in animals, promoting the growth of beneficial bacteria, inhibiting the growth of pathogens, and maintaining the barrier homeostasis [110]. Commonly used feed additives in diets for pigs include organic acids, probiotics, nucleotides, prebiotics, plant extracts, zinc oxide, and copper [14, 111]. Metabolomics has so far been adopted to evaluate the impact of probiotics, bile acid, and fatty acids as feed additives on growth performance and intestinal health by examining the fecal and serum metabolic profiles [112]. Two metabolomics studies on probiotic supplementation in the diet, e.g., *Lactobacillus plantarum* B90, *Saccharomyces cerevisiae* P11, and *Clostridium butyricum* supplementation, have shown a similar effect on the offspring, regardless of whether the probiotic was provided in the diet of the sow or directly in the diet of the weaned piglets. It effectively reduced N-acetylhistamine in feces and colonic digesta of the piglets after weaning [113, 114] (Table 4). N-acetylhistamine is a gut microbial-derived metabolite of histidine metabolism [115], and its level in feces and urine correlates with intestinal disorders and inflammatory bowel disease [116, 117]. Supplementing probiotics in the weaner diet can enhance the ability of the gut microbiota to ferment various substrates from the diet, leading to increased production of fatty acids such as acetic acid, azelaic acid, and dodecanedioic acid in feces, which possess antimicrobial functions and regulate intestinal inflammation [114, 118]. Another strategy is to spray probiotics in the environment [119]. Spraying a mixture of different species of lactic acid bacteria in the living environment of sucking piglets lead to increased tetradecanoic acid and heptadecanoic acid content in serum. Tetradecanoic acid supplementation in the diet has been correlated with stimulation of intestinal morphology with an increased villi length in the small intestine [120].

**Table 4:** The impact of feed additives and early food introduction on the metabolome of piglets and growing pigs

Strategy	Treatments	Duration	Sample	Key results	Affected pathways	References
Probiotics supplement	*Lactobacillus plantarum* ≥ 1 × 10^8^ CFU/mL *Saccharomyces cerevisiae* ≥ 0.2 × 10^8^ CFU/mL Sows’ diet impact on offspring pigs at different ages 92.60 ± 11.76 kg sows	Day 1 of pregnancy until weaning	Colonic content	At 65 days of age: Increased levels of 7-isojasmonic acid, (R)-3-hydroxybutyric acid, 8(R)-hydroperoxylinoleic acid, 9,10–12,13-diepoxyoctadecanoate, and D-glucuronic acid Decreased levels of 9-oxoODE, beta-sitosterol, maleic acid, trioxilin A3, and niacinamide At 95 days of age: Increased levels of 8(R)-hydroperoxylinoleic acid, (R)-3-hydroxybutyric acid, 9,10–12,13-diepoxyoctadecanoate, and D-glucuronic acid Decreased levels of maleic acid and N6, N6, N6-trimethyl-L-lysine At 125 days of age: Increased levels of 1-aminocyclopropanecarboxylic acid and 1-naphthylamine Decreased levels of N-acetylhistamine and tetracosanoic acid	Lysine degradation, non-alcoholic fatty liver disease, insulin signaling, nicotinate and nicotinamide metabolism, pentose phosphate, ABC transporters, non-alcoholic fatty liver disease, rheumatoid arthritis, human papillomavirus infection, biotin metabolism, AMPK signaling pathway, central carbon metabolism in cancer, linoleic acid metabolism, renal cell carcinoma, arginine and proline metabolism, and glucagon signaling pathway	[113]
Probiotics supplement	*Clostridium butyricum* 5 × 10^8^ CFU/kg diet 28 ± 2-day-old weaned piglets’ diet	21 d	Feces	Increased levels of citrulline, acetyl-DL-valine, L-citrulline, 4-hydroxy-L-proline, cimaterol, sebacic acid, suberic acid, azelaic acid, dodecanedioic acid, o-toluic acid, 3-methylglutaric acid, indole-3-carboxylic acid, 4-acetamidobutanoic acid, nonic acid and 2-phenylpropionic acid Decreased levels of L-valine, N-acetylhistamine, phosphatidylinositol lyso 16:0, phosphatidylethanolamine lyso 18:2,* trans*-3-coumaric acid, xanthurenic acid and 2,3-dihydroxybenzoic acid	Arginine and proline metabolism; valine, leucine and isoleucine biosynthesis, phenylalanine metabolism, and fatty acid metabolism	[114]
Probiotics supplement	Multi-strain probiotics 3-week-old weaned piglets’ diet	6 weeks	Feces	Increased levels of acetic acid, proline, uracil and BCAA such as valine, isoleucine	Amino acid metabolism and fatty acid metabolism	[118]
Probiotics supplement	Liquid fermented probiotics Spraying in new-born piglet’ living environment	21 d	Serum	Increased levels of riboflavin, vitamin E, tetradecanoic acid, and heptadecanoic acid	Fatty acid biosynthesis and vitamin digestion and absorption	[119]
Bile acid supplement	Muti-bile acids supplement 27.0 ± 1.5 kg growing-finishing pigs	16 weeks	Serum	Increased levels of 29-demethylgeodisterol-O-sulfite, codonocarpine, inosine, famotidine, isoprothiolane, allopurinol-1-ribonucleoside, guanine, hypoxanthine, arachidonoyl dopamine, dioxibrassinin, callystatin A, glycerophosphocholine, methyl methylthio selenide, and chenodeoxycholic acid 3-sulfate Decreased levels of glycineamideribotide, cyclochlorotine, glucosyloxyanthraquinone, acetylcarnitine, isoeugenitol, dihydrozeatin riboside, auramycinone, frangulin A, bromocriptine	Purine metabolism, ether lipid metabolism, glycerophospholipid metabolism, amino sugar and nucleotide sugar metabolism, and primary bile acid biosynthesis	[121]
Bile acid supplement	Chenodeoxycholic acid 28-day-old weaned piglets	1 month	Serum	Increased levels of 3-(methylthio)-1-propanol, theobromine, 1,2,4-butanetrio, IP7G, N′-formylkynurenine, guanosine, uridine 5-monophosphate, indole-3-acetic acid, hypoxanthine-9-β-D-arabinofuranoside and inosine Decreased levels of 5-chloro-1-methyl-4-nitroimidazole, dulcitol, p–hydroxyphenyl acetic acid, mandelic acid, 5-amino-1-[3,4-dihydroxy-5-(hydroxymethyl)oxolan-2-yl]imidazole-4-carboxamide, 3-hydroxyhippuric acid, isoquinoline, trimethoprim, and N-(3-indolylacetyl)-L-alanine	CDCA supplementation, and purine metabolism, tryptophan metabolism and microbial metabolism	[122]
Dietary supplement	Citrus pulp-integrated diet 25.3 ± 3.0 kg growing pigs	6 weeks	Feces	Increased levels of N-methylschinifoline, maculosidin, kokusaginin, skimmianine, and dihydrosuberenol Decreased levels of 3-indolebutyric acid, methsuximide and isosalsolidine	Calcium signaling pathway and biosynthesis of various alkaloids	[124]
Dietary supplement	2% fermented Chinese herb supplementation 25.75 ± 0.14 kg growing pigs	65 d	Colonic content	Compared 2% fermented Chinese herb supplementation to non-supplemented control: Increased levels of secologanin, 4-amino-4-deoxyarabinose, hexamethylpropylene amine, 5-methyl-5,6,7,8-tetrahydromethanopterin, PS(20:0/22:5(4Z,7Z,10Z,13Z,16Z)), methyl 2-(10-heptadecenyl)−6-hydroxybenzoate, N1-acetyl-tabtoxinine-beta-lactam, 10-propyl-5,9-tridecadien-1-ol, 5-phosphoribostamycin, narbonolide, propionic acid, heptanoic acid, caproic acid, stearidonic acid, and citric acid Decreased levels of 4-(beta-acetylaminoethyl)imidazole, 3-methylbutyl 3-oxobutanoate, N-butyl-N-(4-hydroxybutyl)nitrosamine, 1-(3-aminopropyl)-pyrrolinium, CDP-DG(i-12:0/6 keto-PGF1alpha), PIP(22:4(7Z,10Z,13Z,16Z)/PGF1alpha), 1,6-dihydroxy-5-methylcyclohexa-2,4-dienecarboxylate, (1S,2R,3S,7R,10S,13S,14R)−1-methyl-14-propan-2-yl-2-propyl-12-azapentacyclo[8.6.0.02,13.03,7.07,12]hexadecane, UDP-2,3-diacetamido-2,3-dideoxy-alpha-D-glucuronate, and procaine	Glucosinolate biosynthesis, purine metabolism, the biosynthesis of various antibiotics and alkaloids derived from histidine and purine, histidine metabolism, toluene degradation, and biosynthesis of phenylpropanoids	[126]
Dietary supplement	A blend of medium-chain fatty acids, butyrate, organic acids, and a phenolic compound was supplemented at 0.2% on top of the control diet 25-day-old weaned piglets	28 d	Plasma Small intestine and colonic digesta	Metabolites with increased level in plasma: tryptophan metabolites–indole-3-carboxylic acid, 3-methyloxyindole, 2-methyloxyindole, indole-3-carboxaldehyde and 3-indolepropionic acid at D7 post-weaning; indole-3-carboxylic acid, 3-methyloxyindole, and 2-methyloxyindole were also observed in plasma at D14 post-weaning Metabolites with increased level in small intestine: n-acetylated tryptophan	In plasma: purine metabolism, betaine metabolism, oxidation of branched-chain fatty acids, tryptophan metabolism and riboflavin metabolism In small intestine: tryptophan metabolism	[128]
Early food introduction	Breast milk vs. milk replacer Suckling piglets with milk replacer at d 7 of age were sampled at d 17–21 of age	2 weeks	Jejunal content	Milk replacer compared to breast milk: Increased levels of L-citrulline, betaine, 1,2-dioleoyl-sn-glycero-3-phosphatidylcholine, 5-methylcytosine, cytosine, glycitein, daidzein, N-oleoylethanolamine, L-histidine, acetylcarnitine, 1-myristoyl-sn-glycero-3-phosphocholine, 1-oleoyl-L-.alpha.-lysophosphatidic acid, 2′-deoxyinosine, 1-palmitoyl-sn-glycero-3-phosphocholine, thioetheramide-PC, PC (16:0/16:0), linoleoyl ethanolamide, thymine, arachidonic Acid (peroxide free), 2′-deoxyuridine, genistein, chenodeoxycholate, 4-androsten-17.beta.-ol-3-one glucosiduronate Decreased levels of guanosine, Pro-Glu, 2-hydroxyadenine, N-Acetylmannosamine, N-Acetylneuraminic acid, Pro-Ala, Uridine, N-Acetyl-D-glucosamine, L-arginine, Pro-Thr, Pro-Phe, MG (18:2(9Z,12Z)/0:0/0:0), allopurinol riboside, Ile-Pro, Asp-Leu, Thr-Ala, riboflavin, cholic acid, hypoxanthine, S-methyl-5′-thioadenosine, trimethylamine N-oxide, adenine, 3-methoxy-4-hydroxyphenylglycol sulfate, muramic acid, all *cis*-(6,9,12)-linolenic acid, lumichrome, alpha-D-glucose, D-mannose, pantothenate, L-asparagine, D-lyxose, L-threonine, L-aspartate, inosine	Arginine biosynthesis, pyrimidine metabolism, primary bile acid biosynthesis, valine, leucine and isoleucine biosynthesis, alanine, aspartate and glutamate metabolism, linoleic acid metabolism, taurine and hypotaurine metabolism, pantothenate and CoA biosynthesis, and riboflavin metabolism	[129]
Early food introduction	Creep feeding Suckling piglets	From 7 d after birth until weaning at 28 days of age	Plasma	Increased levels of triglycerides, hippuric acid, phosphatidylcholine, taurine, proline, tyrosine, ornithine, serine, and arginine	Purine metabolism, propanoate metabolism, tricarboxylic acid cycle, pyrimidine metabolism, histidine metabolism, taurine metabolism, nitrogen metabolism and D-glutamine and D-glutamate	[130]

*ABC transporters* ATP-binding cassette transporters, *AMPK* AMP-activated protein kinase, *IP7G* Inositol 1,3,4,5,6-pentakisphosphate guanosine, *PC* Phosphatidylcholine, *MG* Monoacylglycerol

Bile acids, such as chenodeoxycholic acid, hyocholic acid, and glycochenodeoxycholic acid, added to the feed, have been shown to increase hypoxanthine and inosine levels in the metabolic profiles of serum of both 9-week-old growing-finishing pigs and 4-week-old weaned piglets, with a feeding duration of 1 to 4 months depending on the specific experimental aims [121, 122]. The microbial-derived metabolite hypoxanthine has been shown to maintain the barrier function and stimulate the wound healing process in human intestinal epithelial cell lines by modulating the energy metabolism [72]. In addition, inosine can improve intestinal function by modulating host immune and inflammatory responses, and it may protect against colitis, thereby improving gut health [123].

Another feed additive, citrus pulp, added to the diet of growing pigs, has been shown to mainly decrease 3-indolebutyric acid and increase the level of metabolites originating from citrus fruits (N-methylschinifoline, maculosidin, kokusaginin, skimmianine, and dihydrosuberenol) in feces [124]. 3-Indolebutyric acid is a metabolite mainly produced during bacterial protein fermentation in hindgut, which has been associated with impaired gut health at high concentration in the gut [125]. A decrease of 3-indolebutyric in feces could be related to the stimulation of gut health.

A recent study showed potential beneficial effect of dietary supplementation of 2% fermented Chinese herbs in the diet of growing pigs (26 kg) with a feeding duration of 65 d. The feed additive effectively increased metabolites especially fatty acids such as stearidonic acid and propionic acid in the colon [126]. This group of metabolites has been connected to anti-inflammatory and antitumoral effects and with properties in prevention of intestinal diseases [127].

Adding blends of feed additives is strategy to manage post-weaning diarrhea and improve piglet performance. A study found that supplementing a 0.2% blend of medium-chain fatty acids, butyrate, organic acids, and a phenolic compound for 28 d accelerated microbial maturation in post-weaning piglets [128]. This was associated with increased plasma 3-indolepropionic acid and higher levels of N-acetylated tryptophan in the small intestine, suggesting enhanced tryptophan metabolism likely driven by microbiota shifts. As discussed earlier, tryptophan metabolism is important for maintaining gut barrier function.

Besides changes in the three main feed macronutrients, e.g., carbohydrates, protein, and fat, and feed additives, the impact of some feeding strategies on gut health has been evaluated by metabolomics. Early supplementation of milk replacer during the suckling phase has been suggested to stimulate the intestinal function of suckling piglets by increasing L-citrulline, betaine, and daidzein in the jejunal content [129]. Additionally, creep feeding has been shown to increase the levels of triglycerides, hippuric acid, taurine, and phosphatidylcholine in plasma metabolic profiles [130]. Stimulation of taurine metabolism in the small intestine and blood was found in both studies. Dietary taurine supplementation has been shown to improve the colonic epithelial function by increasing tight junction expression in weanling piglets with LPS-induced diarrhea [131]. Time-restricted feeding, which allows the pigs to have access to a set amount of feed at set time points each day, has also been shown as a strategy to improve the gut health and metabolism of growing pigs by increasing colonic hippuric acid, 2-aminobutyric acid, and niacinamide [132]. Niacinamide is one form of vitamin B_3_ important in maintaining cellular energy levels and cell health [133].

Metabolomics has also been used to examine which metabolites correlate highly with high feed efficiency in pigs receiving the same diet. Pigs with high feed efficiency have been shown to have increased 3-(3-hydroxyphenyl) propionic acid in colon content [134], dihydroxycoprostanic acid, dihydrovitamin D_3_, 6-hydroxyhexanoic acid, and m-coumaric acid in feces compared to those with low feed efficiency [135]. Metabolites derived from the gut microbiota, such as propionic acid and m-coumaric acid, have been shown to contribute to immune regulation and the maintenance of cellular redox homeostasis, respectively [136, 137]. This underscores the crucial role of gut microbiota in nutrient absorption and utilization, which can enhance feed efficiency, alter the gut metabolome, and maintain gut health. Therefore, applying metabolomics to identify metabolites associated with high feed efficiency and gut health could uncover novel biochemical mechanisms and predictive biomarkers, ultimately reducing costs and environmental impact while maintaining animal health.

In summary, supplementing diets with feed additives such as probiotics, bile acids, and fatty acids has been shown to result in increased concentration of metabolites considered beneficial for disease prevention and growth performance improvement in piglets and growing pigs. Moreover, the early introduction of milk replacer stimulated the taurine metabolism of suckling piglets, which could improve gut health and cell metabolism. Finally, metabolomics can be a promising approach to evaluate the impact of nutritional strategies on gut health and growth performance by identifying biomarkers related to high feed efficiency.

## Conclusion, limitations, and future perspective

### Conclusion

Metabolomics tools offer great potential to provide mechanistic and molecular insights into complex systems. Most studies included in this review applied untargeted metabolomics due to its ability to screen the metabolic phenotypes of individuals broadly, determine the outcome of different dietary interventions on pig metabolism, and further find biomarkers suitable for the evaluation of gut health status. Recent advancements in metabolomics platforms and annotation methods have expanded the identification of metabolites and improved the interpretation of metabolic pathway modulation, spurring a rise in nutritional metabolomics studies focused on pig health.

From the wide range of the discussed metabolites in the studies reviewed here, those metabolites that seem to contribute to improved gut health and are generally associated with an improved animal performance, include increased levels of azelaic acid, hypoxanthine, stearic acid, arachidonic acid, 9-HODE, and straight-chain saturated fatty acids such as dodecanoic acid (C12), tetradecanoic acid (C14), heptadecanoic acid (C17), and octadecanoic acid (C18), as well as a decreased level of histamine and N-acetylhistamine.

**Fig. 2:**
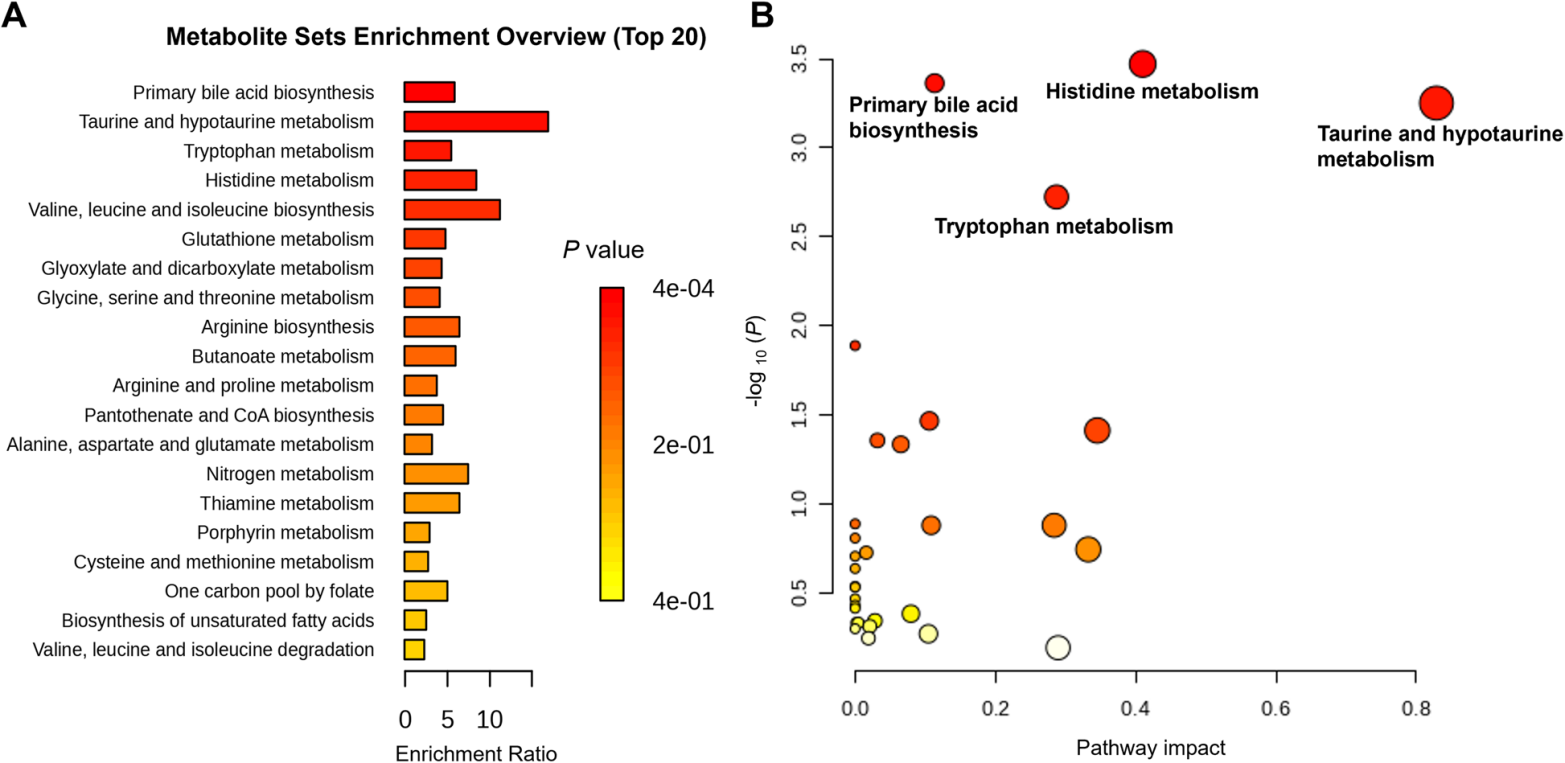
Summary of metabolic pathway enrichment analysis performed in MetaboAnalyst (Version 6.0) using metabolites associated with gut health status in pigs. **A** Metabolic set enrichment analysis identifies the most enriched pathways of altered metabolites under different dietary interventions. **B** Metabolic pathway analysis maps the altered metabolites to pathways in the KEGG pig database. Matched pathways are represented as circles, where color intensity (white to red) indicates increasing statistical significance. The size and color of each circle correspond to the pathway impact value and *P*-value, respectively

To obtain a functional assignment of the metabolites identified under the various dietary interventions reviewed in relation to pig gut health, pathway enrichment analysis was performed using MetaboAnalyst 6.0 (Fig. 2) [138]. This tool maps the groups of compounds identified in this review to known pathways from databases such as KEGG and HMDB. Statistical significance was assessed using the hypergeometric test, and enriched pathways are visualized as bar charts. Additionally, joint pathway analysis is presented through bubble plot, highlighting the most relevant biological processes associated with the identified metabolites. The top four pathways associated with the shifted metabolite sets in the current review are primary bile acid biosynthesis, taurine and hypotaurine metabolism, tryptophan metabolism, and histidine metabolism.

### Limitations and future perspective

There are still challenges and future research perspectives when examining the nutrition-gut interaction by metabolomics. First, targeted metabolomics studies only investigate pre-defined metabolites, leaving many metabolites unstudied. Untargeted metabolomics studies face the challenge of still many unannotated metabolites in the current metabolite libraries. Second, due to inherent limitations such as limited sensitivity, specificity, reproductivity, and rapid metabolite degradation, a single analytical platform cannot provide a global overview of the metabolites produced by a biological system [139]. Future applications and studies can substantially benefit from further technological developments of the metabolomics platform, and furthermore, multi-platform analyses can improve coverage and identification of metabolites related to gut health and performance. Third, a combination of metabolomics results with other omics methods, such as metagenomics, transcriptomics, and proteomics, would provide more insights into the mechanisms underlying biological processes and molecular functions, interactions, and cellular fate [140]. Metabolomics combined with proteomics can reveal the functional state of a biological system and clarify molecular mechanisms of the host-microbe interaction in the gut environment. Besides, integrating metagenomic and metabolomic analysis can help extract a more comprehensive and insightful view of biological systems, which enables researchers to construct complex networks of gene-metabolite interaction [141]. A correlation network model by multi-omics analysis in pig gut can increase the knowledge of microbial molecular functions and could be applied to future feeding strategies. Fourth, the metabolites that may have a beneficial impact on gut health or specific metabolic pathways, as reviewed here, could be utilized as biomarkers to assess the gut health status of pigs or serve as a starting point for identifying feed additives that stimulate pig gut health [72, 142]. This has, for example, been done for dodecanoic acid and hypoxanthine. Dodecanoic acid has been shown to be a potential feed additive improving the gut health of pigs and also mitigating feed pathogens [142]. For hypoxanthine, a wound healing function in EDTA-induced colonic epithelium disruption has been shown, and hypoxanthine is a crucial metabolite that can improve intestinal barrier function and modulate energy metabolism in intestinal epithelial cells; hence, hypoxanthine can be considered as a biomarker to evaluate the health of the colonic epithelium [72].

Lastly, this review shows that different levels and sources of dietary fiber, protein, fat, and feed additives have an impact on the gut metabolome. However, it is generally difficult to conclude which groups of metabolites or pathways can contribute to the best gut health of pigs. More studies are needed to find the optimal levels and types of each ingredient in the diet to stimulate the gut health of pigs. Overall, the age and gender of the pigs, the experimental design, and the sample type differ between the studies, which can affect the metabolites and metabolic pathways influenced by different nutritional approaches. Furthermore, an important issue is to move from correlation between metabolites affected by nutritional interventions and health traits to causality. Additionally, to reduce the use of animals in in vivo studies, in vitro studies can serve as a supplementary screening tool to investigate potential in vivo mechanisms within cell systems. This includes studies utilizing porcine cell lines and porcine organoids to examine the impact of selected metabolites on gut health parameters [143, 144]. Such an approach allows for the screening of metabolites and their combinations before further investigation in in vivo studies, where relevant phenotypes are also included.

## Data Availability

The dataset compiled for this review article is included in the tables and references of this paper.

## Abbreviations

BCAA: Branched-chain amino acid
EDTA: Ethylenediaminetetraacetic acid
GC–MS: Gas chromatography–mass spectrometry
9-HODE: 9-Hydroxy-10E,12Z-octadecadienoic acid
13-HODE: 13-Hydroxy-9Z,11E-octadecadienoic acid
KEGG: Kyoto encyclopedia of genes and genomes
LC–MS: Liquid chromatography–mass spectrometry
LPS: Lipopolysaccharide
NMR: Nuclear magnetic resonance
OSH: O-succinyl-l-homoserine
PWD: Post-weaning diarrhea
SCFAs: Short-chain fatty acids
